# Identification of two organohalide-respiring *Dehalococcoidia* associated to different dechlorination activities in PCB-impacted marine sediments

**DOI:** 10.1186/s12934-017-0743-4

**Published:** 2017-07-24

**Authors:** Andrea Nuzzo, Andrea Negroni, Giulio Zanaroli, Fabio Fava

**Affiliations:** Department of Civil, Chemical, Environmental and Materials Engineering, University of Bologna, Via Terracini 28, 40131 Bologna, Italy

**Keywords:** Polychlorinated biphenyls, Microbial reductive dechlorination, Organohalide respiration, Marine sediments, *Dehalococcoidia*

## Abstract

**Background:**

Microbial reductive dechlorination of polychlorinated biphenyls (PCBs) plays a major role in detoxifying anoxic contaminated freshwater and marine sediments from PCBs. Known members of the phylum *Chloroflexi* are typically responsible for this activity in freshwater sediments, whereas less is known about the microorganisms responsible for this activity in marine sediments. PCB-respiring activities were detected in PCB-impacted marine sediments of the Venice Lagoon. The aim of this work was to identify the indigenous organohalide-respiring microorganisms in such environments and assess their dechlorination specificity against spiked Aroclor™ 1254 PCBs under laboratory conditions resembling the in situ biogeochemistry.

**Results:**

High PCB dechlorination activities (from 150 ± 7 to 380 ± 44 μmol of chlorine removed kg^−1^ week^−1^) were detected in three out of six sediments sampled from different locations of the lagoon. An uncultured non-*Dehalococcoides* phylotype of the class *Dehalococcoidia* closely related to *Dehalobium chlorocoercia* DF-1, namely phylotype VLD-1, was detected and enriched up to 10^9^ 16S rRNA gene copies per gram of sediment where dechlorination activities were higher and 25-4/24-4 and 25-2/24-2/4-4 chlorobiphenyls (CB) accumulated as the main tri-/dichlorinated products. Conversely, a different phylotype closely related to the SF1/m-1 clade, namely VLD-2, also enriched highly where lower dechlorination activity and the accumulation of 25-3 CB as main tri-chlorinated product occurred, albeit in the simultaneous presence of VLD-1. Both phylotypes showed growth yields higher or comparable to known organohalide respirers and neither phylotypes enriched in sediment cultures not exhibiting dechlorination.

**Conclusions:**

These findings confirm the presence of different PCB-respiring microorganisms in the indigenous microbial communities of Venice Lagoon sediments and relate two non-*Dehalococcoides* phylotypes of the class *Dehalococcoidia* to different PCB dechlorination rates and specificities.

**Electronic supplementary material:**

The online version of this article (doi:10.1186/s12934-017-0743-4) contains supplementary material, which is available to authorized users.

## Background

Polychlorinated biphenyls (PCBs) are a family of 209 congeners composed by a biphenyl ring carrying one to ten chlorine substitutions. After more than 30 years of worldwide ban, PCBs are still widespread environmental contaminants [1] and are included in the list of persistent organic pollutants (POPs) targeted for elimination by the Stockholm Convention [2]. The most relevant long-term reservoirs of PCBs released into the environment are aquatic sediments, where PCBs can exert broad toxicity to wildlife [3] and enter the food chain [1, 4, 5]. Under anoxic conditions, generally occurring in such sediments few centimetres below the surface, highly chlorinated PCB congeners can be transformed by microbial activities, which sequentially remove chlorine atoms from the pollutants, making them be more susceptible to aerobic oxidative biodegradation and often less toxic and less prone to bioaccumulate than parent compounds [6, 7]. Such activity is called microbial reductive dechlorination and represents a promising process for the sustainable remediation of contaminated sediments, currently managed through expensive and highly-impacting dredging operations or capping [8, 9].

The occurrence of microbial PCB-dechlorinating activity in freshwater and estuarine sediments was documented in several laboratory sediment cultures and sediment-free cultures developed with synthetic media [7, 10–16]. The process is mainly mediated by members of the phylum *Chloroflexi* and, less frequently, *Firmicutes*, which use PCBs as terminal acceptor for their electron transport chain [7, 10, 17, 18]. Among the organohalide-respiring *Chloroflexi*, members of the genus *Dehalococcoides* were predominantly associated to PCB dechlorination in freshwater systems [10, 12, 14, 16, 19, 20] and more rarely in estuarine environments [21, 22]. On the other hand, *Dehalococcoides*-like *Dehalococcoidia* belonging to the o-17/DF-1 clade have been more frequently linked to PCB dechlorination activity in estuarine environments [21, 23–25], where salinity and sulfate concentrations may shift over time and space between freshwater and marine conditions [26]. Moreover, a number of pure cultures of *Dehalococcoides* species isolated from freshwater environments were recently shown to dechlorinate a wider range of organohalides, including PCBs [19, 27–29], in which some reductive dehalogenases were characterized in terms of regio specificities towards PCB congeners [27, 30]. Notably, a non-*Dehalococcoides* strain, *Dehalobium chlorocoercia* DF-1, is the sole *Chloroflexi* PCB respiring isolate obtained so far from estuarine sediments [24, 31].

Microbial dechlorination has been less investigated in marine conditions [17, 32–38] where higher salinity and sulfate concentrations select for different microbial taxa compared to estuarine or freshwater environments [39]. Limited information is also available on the PCB respiring *Chloroflexi* in marine environments. In sediments of the Gulf of Taranto (Mar Piccolo, Ionian Sea, Italy), the enrichment of *Dehalococcoides mccartyi* and its PCB reductive dehalogenase-coding genes was reported during PCB dechlorination [40, 41]. Conversely, non-*Dehalococcoides Dehalococcoidia* were detected in sediments of the Venice Lagoon after sub-culturing of the indigenous community in the presence of exogenous PCBs [34, 42] as well as after biostimulation with zerovalent iron nanoparticles [43], suggesting that this group of organohalide respiring *Chloroflexi* might be relevant for the bioremediation of PCB-contaminated marine sediments. The aims of this work were: (i) to further assess the dechlorination potential, in terms of occurrence, dechlorination rate and specificity, of the indigenous microbial communities of the PCB-contaminated sediments in the Porto Marghera area (Venice Lagoon) and (ii) to assess the role of *Dehalococcoidia* in the process in terms of dehalogenation specificity and growth during organohalide respiration.

## Results and discussion

### PCB dechlorination

**Fig. 1:**
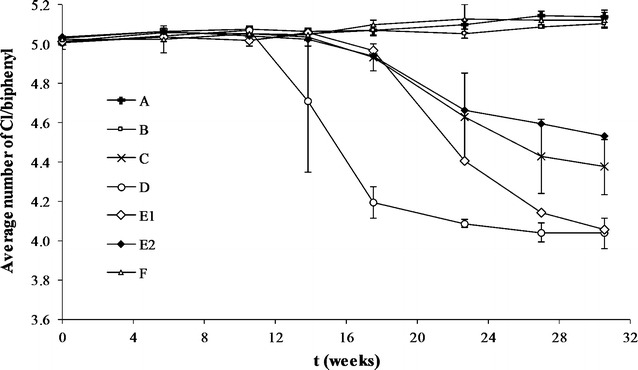
Reductive dechlorination of Aroclor 1254 PCBs in the biologically active sediment cultures. Values (average number of chlorine substituents/biphenyl molecule) are the mean (±SD) of replicate cultures, except for E1 and E2 replicate cultures

Six different sediments from the Venice Lagoon (namely, A, B, C, D, E and F), historically contaminated by PCBs at low concentrations (Additional file 1: Table S1) were cultivated in biogeochemical conditions resembling those occurring in situ and spiked with Aroclor 1254 (see “Methods”). Neither autoclaved controls nor biologically active cultures of sediment A, B and F exhibited PCB dechlorination activities throughout incubation (31 weeks). Conversely, PCB dechlorination started after 11 weeks of incubation in sediment D cultures and after 14 weeks of incubation in sediment C and E cultures (Fig. 1). The onset and extent of PCB dechlorination (mol% of Cl removed) was not related to the pre-existing concentration (Pearson coefficient 0.59) and distribution of congeners (Pearson coefficients in the range 0.04–0.52 for different homologue groups) of PCBs associated with the sediment matrix (Additional file 1: Table S1). This might be due to possible differences in the sediment indigenous microbial community or in some unknown sediment feature (i.e. total organic carbon available, trace metal contaminants, etc.), that might have limited the growth and activity of some fractions of the microbial community, including PCB dechlorinators. Dechlorination proceeded at maximum rates of 380 ± 44 and 200 ± 31 μmol of chlorine removed kg^−1^ week^−1^ in sediment D and C cultures, respectively. Replicates of sediment E cultures exhibited remarkably different PCB dechlorination rates, being in replicate 1 (herein after referred to as sediment E1 culture) comparable to sediment D cultures and in replicate 2 (herein after referred to as sediment E2 culture) comparable to sediment C cultures (325 ± 21 and 150 ± 7 μmol of chlorine removed kg^−1^ week^−1^, respectively). Similarly, more extensive PCB depletions occurred in sediment D and E1 cultures (84 and 77 mol% of penta- to octa-CBs removed, respectively), compared to sediment C and E2 cultures (62 and 51 mol% of penta- to octa-CBs removed, respectively).

**Fig. 2:**
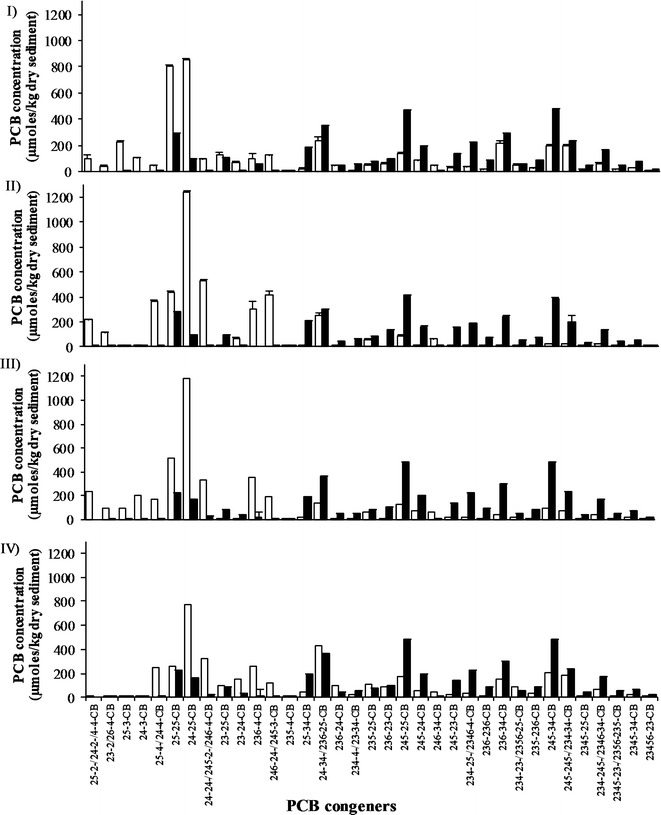
Concentration (µmoles kg^−1^) of PCB congeners in the biologically active (*white bars*) and sterile (*black bars*) sediment C, D, E1 and E2 cultures (*panels I*, *II*, *III* and *IV*, respectively) after 31 weeks of incubation. Only congeners representing more than 1% w/w of the total PCBs are reported. Values are the mean (±SD) of replicate cultures, except for E1 and E2 replicate cultures

The accumulation of several tetra- and tri-chlorinated congeners was observed in all PCB dechlorinating cultures (Fig. 2), leading to the decrease of the initial average number of chlorines per biphenyl ring (5.1) to 4.0 in sediment D and E1 cultures and to 4.4 and 4.5 in sediment C and E2, respectively (Fig. 1).

**Fig. 3:**
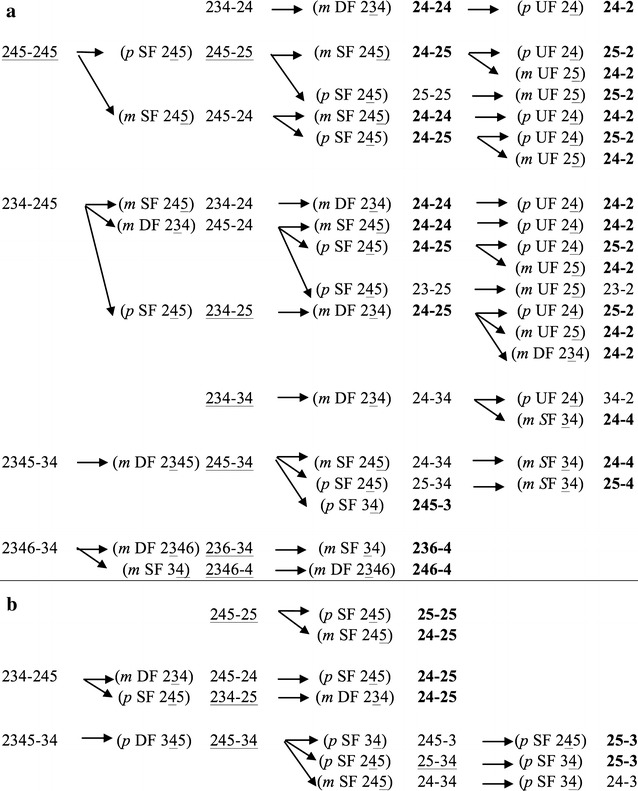
Possible dechlorination patterns of the mostly depleted hexa- and penta-chlorinated congeners leading to the most prominently accumulated tetra- and tri-chlorinated congeners, in sediment D (*panel* **a**) and sediment C (*panel* **b**) cultures. Congeners (either eluting alone or co-eluting with others) of the original Aroclor 1254 mixture that were more extensively dechlorinated are underlined, whereas congeners (either eluting alone or co-eluting with others) that accumulated in higher amounts (more than 200 µmol kg^−1^ after 31 weeks of incubation) are in *bold* characters. For each dechlorination step, the following information is reported in* parenthesis*: position of the removed chlorine (*m*: *meta*, *p*: *para*), presence of flanking chlorines (*DF* double flanked, *SF* single flanked, *UF* unflanked) and chlorophenyl ring dechlorinated (removed chlorine *underlined*)

The main dechlorination products that accumulated at the end of the incubation in sediment D were, in decreasing order of µmoles kg^−1^ accumulated since the beginning of incubation: 24-25 CB (1074 µmol kg^−1^) > 24-24/245-2/246-4 CB (510 µmol kg^−1^) > 246-24/245-3 CB (405 µmol kg^−1^) > 25-4/24-4 CB (356 µmol kg^−1^) > 234-2/236-4 CB (302 µmol kg^−1^) > 25-25 CB (214 µmol kg^−1^) > 25-2/24-2/4-4 CB (213 µmol kg^−1^) > 23-2/26-4 CB (116 µmol kg^−1^) (Fig. 2). No congener specific mass balance could be performed, due to the co-elution of several low-chlorinated products. However, considering all the possible pathways leading from the mostly depleted congeners (either eluting alone or with others, i.e., 245-34, 245-25, 236-34 and 245-245/234-34 CB) to the main dechlorination products that accumulated at the end of the incubation (Fig. 2), the most recurrent dechlorination activities potentially occurring, in terms of targeted position and substitution pattern of the chlorophenyl ring, could be identified (Fig. 3a). In particular, *meta* dechlorination of double flanked chlorines on 2345, 2346 and 234 chlorophenyl rings appears very common to several possible pathways, along with *meta* and *para* dechlorination of single flanked chlorines in 245 and, to less extent, 34 chlorophenyl rings. Dechlorination of unflanked *meta* and *para* chlorines in 24 and 25 chlorophenyl rings also occurred, leading through different possible pathways to the accumulation of 24-2 and 25-2 congeners. This dechlorination activity was probably slower or less efficient than others, as indicated by the remarkably lower accumulation of 24-2 and 25-2 congeners compared to their parent tetra-chlorinated CBs (24-24, 24-25) and the lack of 2-2 congener accumulation. The removal of unflanked *meta* and *para* chlorines represents an important dechlorination activity to achieve extensive decontamination of sediments impacted by complex PCB mixtures, as it allows the complete elimination of *meta* and *para* chlorines, rarely observed in marine sediments but more frequently in freshwater or estuarine sediments [21, 22, 44, 45].

Conversely, 24-25 CB (760 µmol kg^−1^) and 25-25 CB (511 µmol kg^−1^) were the most accumulated dechlorination products in sediment C, where formation of 25-3 CB (227 µmol kg^−1^) and 24-3 CB (105 µmol kg^−1^) was also detected, along with a much lower accumulation of 246-24/245-3 CB (117 vs 405 µmol kg^−1^), 25-2/24-2/4-4 CB (100 vs 213 µmol kg^−1^), 24-24/245-2/246-4 CB (84 vs 510 µmol kg^−1^) and 25-4/24-4 CB (42 vs 356 µmol kg^−1^) compared to sediment D (Fig. 2). Considering all the possible dechlorination pathways leading from the mostly depleted congeners of the Aroclor 1254 mixture (i.e., 245-25, 245-34 and 234-245/2346-34 CB) to the dechlorination products that accumulated in higher amounts (Fig. 3b), the most recurrent dechlorination activity in sediment C cultures was directed towards the *para* position, in particular of single flanked chlorines in 34, 234 and 245 chlorophenyl rings. In addition, some *meta* dechlorination, mainly of single flanked chlorines in 245 chlorophenyl rings and, to less extent, of double flanked chlorines in 234 chlorophenyl rings, may have occurred in some dechlorination pathway (Fig. 3b). Overall, while *para* dechlorination of single flanked chlorines in 245 and 34 chlorophenyl rings and *meta* dechlorination of single flanked chlorines in 245 chlorophenyls are common to potential pathways identified in both sediment C and sediment D cultures, only potential pathways identified for sediment D culture involve also recurrent dechlorination of *meta* double-flanked chlorines on 2345, 2346 and 234 chlorophenyl rings and of *meta* and *para* unflanked chlorines on 24 and 25 chlorophenyl rings. These differences between sediment D and sediment C cultures suggest that PCB dehalogenating bacteria having different dechlorination specificities were enriched in the 2 cultures.

Sediment E1 culture exhibited a dechlorination pattern similar to that of sediment D, being 24-25 CB (879 µmol kg^−1^), 25-25 CB (514 µmol kg^−1^), 236-4 CB (348 µmol kg^−1^), 25-2/24-2/4-4 (230 µmol kg^−1^) and 24-24/245-2/246-4 CB (225 µmol kg^−1^) the main dechlorination products accumulated at the end of incubation (Fig. 2). Few common features to sediment C culture were however also observed, such as the detection of 24-3 CB (205 µmol kg^-1^) and 25-3 CB (88 µmol kg^−1^) (Fig. 2). A dechlorination pattern apparently similar to that of sediment D culture was observed also in sediment E2 culture, although the less extensive dechlorination observed limits the possibility to compare it with the other sediment cultures (Fig. 2).

### Sulfate-reducing and methanogenic activities

Since sulfate-reducing and methanogenic bacteria are the main competitors for electron donors of organohalide respirers in anaerobic sediments, sulfate-reduction and methanogenesis were monitored over incubation to assess the presence of possible inhibitory effects of these metabolisms on the occurrence of PCB respiration. Sulfate concentrations spanned from 27 ± 1 to 39 ± 1 mM at the beginning of the incubation. Sulfate consumption was first detected after a lag phase of 6–11 weeks of incubation except in sediment C cultures, where it started immediately and consumed more than 40% of sulfate in the first 6 weeks (Additional file 2: Figure S1). Complete sulfate depletion was achieved in sediment D and sediment C cultures after 11 weeks of incubation, i.e. before the onset of the dechlorination process, but also in sediment A and F cultures, where no PCB dechlorination occurred (Additional file 2: Figure S1). Slower sulfate reduction took place in the non-PCB-dechlorinating culture B, where sulfate was completely reduced before week 23, and in PCB-dechlorinating sediment E1 and E2 cultures, where complete sulfate depletion occurred at weeks 14 and 23 (Additional file 2: Figure S1). No correlation was therefore observed between the extent and rate of sulfate reduction and the occurrence, extent and rate of PCB dechlorination.

Negligible methane production (lower than 0.2 mmol over 31 weeks of incubation) was observed in all sediment cultures, except for the non-PCB-dechlorinating cultures of sediment A, where 56 ± 4 mL (i.e. 2.5 ± 0.2 mmol) of methane were produced (Additional file 3: Figure S2). Therefore, methane production was not related to the dechlorination activities detected, as previously observed in the same area [35, 42].

### Changes in bacterial communities and identification of PCB dechlorinating bacteria

**Fig. 4:**
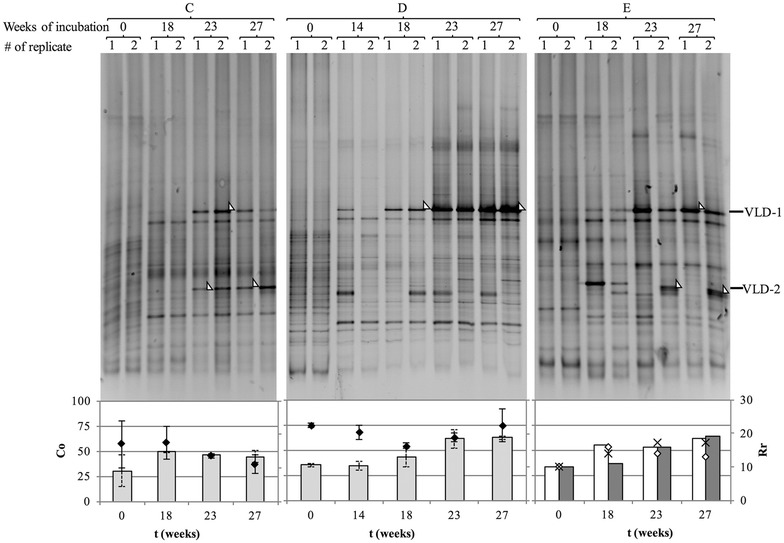
Dynamics of the total bacterial community of the PCB dechlorinating cultures from sediments *C*, *D* and *E* (*left*, *centre* and *right*, respectively). *Upper part* PCR-DGGE analysis of the 16S rRNA genes. *Arrows* indicate excised bands corresponding to phylotypes VLD-1 and VLD-2. *Lower part* community organization (Co, expressed as Gini coefficient times 100) (*bars*) and richness (Rr) (*diamonds*) of the total bacterial communities. Values are the mean (±SD) of replicate cultures, except for E1 and E2 replicate cultures (*empty diamonds* and *stars* respectively)

**Fig. 5:**
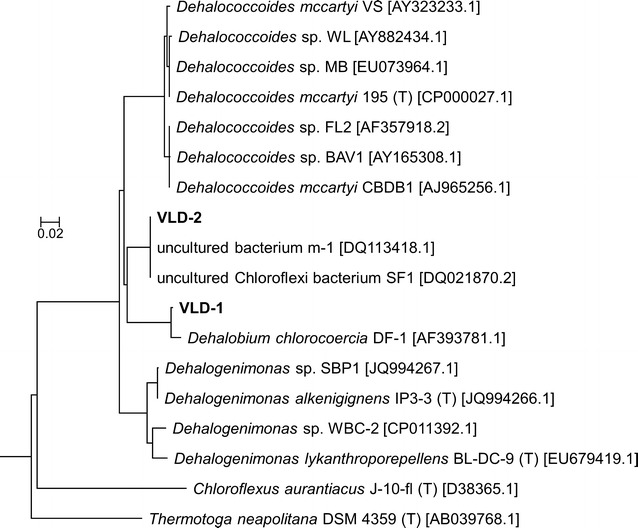
Phylogenetic placement of VLD-1 and VLD-2 within the class *Dehalococcoidia* of the phylum *Chloroflexi*. The tree was created using the Tree Builder tool based on the Neighbour Joining method available at the Ribosomal Database Project (RDP, release 11, http://rdp.cme.msu.edu)

PCR followed by denaturing gradient gel electrophoresis (PCR-DGGE) of the 16S rRNA genes was carried out on sediment C, D and E cultures at the beginning of incubation and during PCB dechlorination to investigate the changes of the indigenous microbial community concurrent to PCB dechlorination. Since very low methanogenic activity occurred in PCB dechlorinating cultures, the analysis was limited to *Bacteria* only (Fig. 4). PCR-DGGE profiles were analysed to investigate changes in richness and community structure of the communities (Fig. 4). While different trends were observed in the community richness of the dechlorinating cultures, a similar increase in the community organization index (Co, e.g., in the Gini coefficient from approximately 30 to 50% or above) occurred after the onset of PCB dechlorination in all dechlorinating cultures. This indicates a shift of the community towards a limited number of specific phylotypes, some of which were potentially involved in the dechlorination process. Indeed, a single band (phylotype VLD-1) became prominent in sediment D and E1 cultures after the onset of PCB dechlorination, while an additional band (phylotype VLD-2) was enriched in sediment C and E2 cultures. The 16S rRNA gene of phylotype VLD-1 had 99.8% sequence identity (514 over 515 base pairs) to that of the uncultured bacterial clones TfC20H76 and TfC20H31, previously enriched in PCE dechlorinating cultures from marine tidal flat sediments [46], and 98.8% sequence identity (505 over 516 base pairs) to that of the known PCB dechlorinating *Chloroflexi* bacterium *Dehalobium chlorocoercia* DF-1 isolated from estuarine sediments [24]. Conversely, the 16S rRNA gene of phylotype VLD-2 had 100% sequence identity (over 471 base pairs) to the uncultured *Chloroflexi* bacteria SF1 and m-1, both identified as PCB dechlorinators in estuarine sediments [21, 47], and to phylotype VL-CHL1 (over 496 base-pairs), that was previously enriched in a PCB dechlorinating culture obtained from a sediment of the Venice Lagoon [34]. The phylogenetic placement of VLD-1 and VLD-2 within the class *Dehalococcoidia* of the phylum *Chloroflexi* is shown in Fig. 5.

**Fig. 6:**
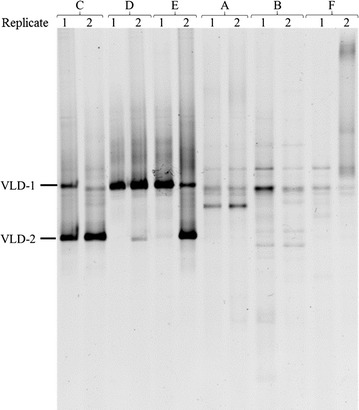
PCR-DGGE analysis of the *Chloroflexi* bacterial community after 27 weeks of incubation in the PCB dechlorinating (*C*, *D*, *E*) and non-dechlorinating cultures (*A*, *B*, *F*). *Arrows* indicate excised bands corresponding to phylotypes VLD-1 and VLD-2

PCR-DGGE analysis of the 16S rRNA genes carried out with primers specific for *Chloroflexi* after 27 weeks of incubation (Fig. 6) revealed the same selective enrichment of the VLD-1 and VLD-2 phylotypes in the PCB dechlorinating cultures, as well as their absence in cultures lacking PCB dechlorination activity. This confirmed that no other *Chloroflexi* came to relevance in any of the PCB dechlorinating cultures when the dechlorination process was almost completed (week 27) and suggested that the selective enrichment of phylotypes VLD-1 and VLD-2 is associated to the different PCB dehalogenation activities observed.

To confirm that growth of the two *Chloroflexi* phylotypes is linked with different dechlorination activities, qPCR was used to quantify their 16S rRNA genes and their relative abundance as a percentage the total 16S rRNA genes (Table 1). Phylotypes VLD-1 and VLD-2 were present in the range of 10^6^–10^7^ copies per gram of sediment in all PCB dechlorinating cultures at the beginning of the incubation, representing a minor share of the total bacterial community (typically less than 1%), and increased throughout dechlorination. The growth yield of *D. mccartyi* on Aroclor 1254 PCBs in marine sediments (i.e., 4.94 × 10^7^ copies per µmol Cl^−^ removed) [40], was used to calculate the expected increases in copy numbers of PCB-dechlorinating *Dehalococcoidia*, which were compared to the copy number increase of VLD-1 and VLD-2 phylotypes (Table 1). Remarkably, in all cultures the observed cumulative increase of VLD-1 and VLD-2 was comparable or higher than the expected one, albeit with differences between the two phylotypes, thereby providing supporting evidence that these two phylotypes grew as PCB respirers. In sediment D and E1 cultures, the 16S rRNA copy numbers of phylotype VLD-1 increased 100-fold after 27 weeks of incubation, to relative abundances of 15.2 ± 1.0 and 55.5 ± 2.6%, respectively. Phylotype VLD-2 16S rRNA gene copy numbers did not increase significantly in sediment D cultures and eventually decreased in sediment E1 cultures after 27 weeks of incubation. On the contrary, in sediment C cultures, phylotype VLD-2 16S rRNA gene copy numbers increased 500-fold, up to 7.4 × 10^9^ ± 1.2 × 10^9^ per gram of sediment after 27 weeks of incubation (accounting for 55.9 ± 5.2% of the bacterial 16S rRNA genes). VLD-1 16S rRNA gene copy numbers also notably increased 100-fold over the course of incubation, but copy numbers were more than 10 times lower than VLD-2 16S rRNA gene copies from the beginning to the end of incubation. Finally, in sediment E2 cultures, both VLD-1 and VLD-2 16S rRNA copy numbers increased from 10^6^ to 10^8^ per gram of sediment, with no significant difference between the two. Therefore, a selective enrichment of phylotype VLD-1 throughout incubation could be detected both in sediment D and E1 cultures, where phylotype VLD-2 16S rRNA gene copy numbers either remained constant or decreased. Provided that these cultures exhibited the same dechlorination specificity and rate, it is reasonable to conclude that VLD-1 was the predominant organohalide respirer in these cultures and could thus be linked to the more rapid and extensive dechlorination activity involving *meta* double flanked chlorines, *meta* and *para* single flanked chlorines and, to less extent, unflanked *meta* and *para* chlorine (Fig. 3a). The enrichment of VLD-2 phylotype in sediment C and E2 cultures was less selective, since in both cases it was accompanied by a simultaneous enrichment of phylotype VLD-1. However, VLD-2 copy numbers in sediment C cultures were always more than tenfold higher than VLD-1 copy numbers, indicating that it nevertheless constituted a major share of the dehalogenating bacteria, whereas similar initial and final copy numbers were observed for VLD-1 and VLD-2 in sediment E2. Therefore, the peculiar features of the dechlorination activities exhibited by sediment C cultures, which differ from sediment D and E1 cultures (i.e. single or double flanked *para* chlorines and, to less extent, single flanked *meta* chlorines removal) (Fig. 3b), could be ascribed to phylotype VLD-2. The presence of some *meta* dechlorination of double flanked chlorines in sediment C culture, on the other hand (Fig. 2a), could be explained by the concurrent, though more limited, enrichment of VLD-1 phylotype. The concomitant and equivalent enrichment of VLD-1 and VLD-2 phylotypes in sediment E2 cultures is consistent with the observation of a dechlorination pattern that is partially similar to that of sediment D and E1 cultures and partially to that of sediment C cultures. Finally, both PCB-dechlorinating phylotypes occurred at very low relative abundances in all cultures at the beginning of incubation, but their differential enrichment and the subsequent different dechlorination activity exhibited suggests that some unknown features of the sediment, or differences in the non-organohalide-respiring fraction of their indigenous microbial community, might preferentially favour the growth of one PCB respiring bacterium over the other. These findings overall confirm the role of non-*Dehalococcoides Dehalococcoidia* in the reductive dechlorination of PCBs in marine sediments of Venice Lagoon, further supporting the relevance of this group of microorganisms for the bioremediation of marine environments contaminated by organohalides.

**Table 1:** 16S rRNA gene copy numbers and relative abundance of phylotypes VLD-1 and VLD-2

	Time (weeks)	Organic chlorine (µmol/gdw)	Expected increase^a^	VLD-1	VLD-2	VLD-1	VLD-2
			(16S rRNA gene copies per gram of sediment)	(16S rRNA gene copies per gram of sediment)		Relative abundance (%)	
C	0	23.2 ± 0.4	–	2.1E+6 (±6.7E+5)	1.5E+7 (±9.8E+6)	0.1 ± 0.0	0.4 ± 0.3
	27	20.4 ± 1.5	1.4E+8 (±9.2E+7)	3.5E+8 (±2.7E+8)	7.4E+9 (±1.2E+9)	2.5 ± 1.9	55.9 ± 5.2
D	0	23.2 ± 0.7	–	9.2E±7 (±9.5E+7)	1.2E+8 (±7.5E+6)	0.6 ± 0.6	0.8 ± 0.1
	27	18.6 ± 0.3	2.3E+8 (±5.2E+7)	2.0E+9 (±7.1E+8)	3.1E+8 (±2.1E+8)	15.2 ± 1.0	2.1 ± 0.9
E1	0	23.3 ± 0.1	–	6.7E+6 (±1.7E+6)	1.3E+7 (±6.3E+6)	1.2 ± 0.4	2.4 ± 1.3
	27	19.4 ± 0.2	1.9E+8 (±1.7E+7)	4.8E+8 (±2.2E+7)	1.9E+5 (±4.6E+4)	55.5 ± 2.6	0.0 ± 0.0
E2	0	23.3 ± 0.9	–	2.6E+6 (±4.2E+6)	5.3E+6 (±3.3E+6)	0.3 ± 0.1	0.5 ± 0.1
	27	21.0 ± 0.9	1.1E+8 (±9.2E+7)	3.1E+8 (±3.2E+7)	1.1E+8 (±1.9E+6)	58.2 ± 4.8	22.0 ± 1.7

Organic chlorine, expected increase of 16S rRNA gene copies of PCB respirers, observed 16S rRNA genes copy numbers per gram of sediment and relative abundance (vs total number of bacterial 16S rRNA genes) of phylotypes VLD-1 and VLD-2 at the beginning and after 27 weeks of incubation (27 week). Values are the mean (±SD) of triplicate analysis performed on metagenomic DNA extracted from each replicate culture with primer pairs targeting 16S rRNA genes of individual phylotypes (VLD-1, VLD-2) and total bacteria

^a^The expected increase of 16S rRNA gene copy numbers of PCB respirers was calculated from the organic chlorine removed over incubation using the growth yield of *D. mccartyi* on PCBs reported in Matturro et al. [40]

## Conclusions

Three out of six marine sediments used in the study showed PCB-dechlorination activities under laboratory conditions resembling the in situ biogeochemistry, suggesting that a potential for dehalogenation is present, although not ubiquitously, in the microbial communities of the Porto Marghera area of Venice Lagoon. Two non-*Dehalococcoides* phylotypes of *Dehalcoccoidia*, closely related to other PCB-respiring microorganisms previously identified in estuarine sediments, were associated to two distinct dechlorination activities. Among these, phylotype VLD-1 is capable of unflanked *meta* and *para* chlorines removal, and thus potentially able to achieve extensive decontamination and detoxification of sediments impacted by complex PCB mixtures. These non-*Dehalococcoides Dehalococcoidia* are therefore candidate targets for further enrichment and isolation efforts, aiming at the production of PCB dechlorinating inocula for bioaugmentation purposes, and/or for biostimulation approaches to promote the decontamination of sediments in the Venice Lagoon and other PCB impacted marine areas.

## Methods

### Venice Lagoon sediments

Six sediments (A, B, C, D, E, and F) from different locations of the first industrial area of Porto Marghera (Venice Lagoon, Italy) were used in this study, along with the seawater collected from the same area. Sediments were impacted by PCBs in the range 0.2 (sediment F)–3.3 (sediment D) mg kg^−1^ consisting predominantly of highly chlorinated congeners (i.e. sediments B and D) but also with considerable percentages of medium–low chlorinated ones (i.e., sediment A) (Additional file 1: Table S1).

### Preparation and sampling of sediment cultures

A set of four 100 mL anaerobic slurry cultures (duplicate biologically active and sterile controls) was prepared for each sediment anaerobically using site water for sediment re-suspension (20% dry w/v) under nitrogen atmosphere [33]. Given the limited number and low concentration of PCB congeners occurring in each sediment (Additional file 1: Table S1), sediment cultures were spiked with Aroclor 1254 (20 g L^−1^ stock solution in acetone) at a final concentration of 1 g of PCBs kg^−1^, to favour the enrichment of sediment indigenous PCB-respiring bacteria and better assess their PCB dechlorination potential. Sediment cultures were incubated statically in the dark at 28 °C for 31 weeks and periodically sampled according to the procedure described by [12] to analyse: (i) the volume and composition of the biogas (i.e., methane and CO_2_), (ii) the congeners and concentration of PCBs in the sediment, (iii) the concentration of sulfate in the water phase and (iv) the structure and composition of the microbial community.

### PCB extraction and analytical procedures

PCBs were batch extracted in duplicate from each replicate culture according to procedures described elsewhere [33]. GC-ECD analyses of extracted PCBs were performed under the analytical conditions described in literature [32]. Qualitative analysis was performed by comparing retention times (relative to octachloronaphtalene) of parent PCBs and their dechlorination products with those of PCBs occurring in Aroclor 1242 and Aroclor 1254 standard mixtures (Ultra Scientific Italia, Bologna). Quantitative analyses were performed using the GC-ECD response factor of each PCB, obtained from linear five-points calibration curves Aroclors (in the range 1.0–50.0 mg L^−1^) and the weight percentage of each congener occurring in Aroclors reported elsewhere [48]. PCB concentrations (μmoles kg^−1^, referred to the sediment dry weight), the average number of Cl per biphenyl and dechlorination rates (μmoles of Cl released kg^−1^ week^−1^, referred to the sediment dry weight) were calculated assuming co-eluting congeners to be present in equal proportions as described in previous works [42].

Biogas production was measured at each sampling with an airtight glass syringe, while its composition was determined via µGC-TCD as described previously [49]. Sulfate concentration in the water phase was determined with IC-CD as described in [32].

### Community analysis by PCR-DGGE of the 16S rRNA gene

Total DNA was extracted from the wet sediment (approximately 250 mg) recovered from the centrifugation of 2 mL slurry samples at 10,000×*g* for 10 min with the UltraClean Soil DNA kit (MoBio Laboratories, Carlsbad, CA, USA) according to the protocol “for maximum yields” provided by the manufacturer preceded by treatment with Proteinase K and Lysozyme as described elsewhere [42]. Total DNA was quantified using Qubit^®^ dsDNA HS Assay Kit with a Qubit 3.0 fluorimeter, following the manufacturer’s specifications.

For DGGE analysis, 16S rRNA genes of the bacterial community were PCR-amplified with the GC-clamped primer GC-357f and primer 907r [39], while 16S rRNA genes of putative dechlorinating *Chloroflexi* were PCR amplified with the GC-clamped primer GC-348f and primer 844r [47]. PCR reactions were performed as described in previous works [27]. DGGE of PCR products (approximately 400 ng DNA per lane) were performed in 7% (w/v) polyacrylamide gels at 55 V for 16 h. Denaturing gradients from 40 to 60% denaturant and from 45 to 55% denaturant were used to resolve total Bacteria and *Chloroflexi*-specific amplicons, respectively. Digital images of gels were captured in UV transillumination after staining with SYBR Green I.

Community richness (Rr) and community organization (Co) indexes were calculated from DGGE image analysis as described in literature [50–52]. In particular, the range-weighted richness was calculated from the total number of bands in the pattern and the denaturing gradient comprised between the first and the last band of the pattern, whereas the community organization was derived from Pareto-Lorenz (PL) evenness curves and the respective Gini coefficient.

Most prominent DGGE gel bands were cut, DNA eluted overnight at 4 °C in sterile water, re-amplified and resolved again in DGGE as described above, before amplification with non-GC-clamped primers. Amplicons were finally purified in the presence of 10 U of ExoI and 1 U of FastAP enzymes (Thermo Scientific Italia s.r.l, Milan, Italy) at 37 °C for 15 min before sequencing with the corresponding forward primer. Sequencing was performed by BMR Genomics (Padova, Italy). Each 16S rRNA gene sequence obtained (∼500 bp) was aligned to the bacterial 16S rRNA database of the Ribosomal Database Project (RDP, release 11, http://rdp.cme.msu.edu) and the closest relative and closest cultured relative retrieved with the Seqmatch tool. The phylogenetic affiliation of each sequence was obtained from the same website with the Classifier tool. Nucleotide sequences were deposited in the GenBank database under the Accession Numbers KR013281 and KR013282.

### Quantification of target *Dehalococcoidia* by qPCR

Primer pairs were designed for the specific amplification of two partial 16S rRNA gene sequences detected in the PCB dechlorinating cultures by DGGE using the bacterial primers pair 357f/907r (see Results section). Primers were designed with the Primer-BLAST website (http://www.ncbi.nlm.nih.gov/tools/primer-blast) [53], using each target sequence as PCR template, a primer melting temperature from 58 to 61 °C, the Refseq RNA database for specificity checking, PCR product Tm between 80 and 90 °C, optimum at 80 °C. The specificity of each candidate primer pairs was checked in silico with the ProbeMatch tool available at the RDP II web site; primer pairs having the highest in silico specificity were checked for cross-specificity in vitro in end-point PCR and qPCR assays (see details below).

The gene copies of the target *Chloroflexi* phylotypes and the total bacterial 16S rRNA genes were quantified through qPCR using a StepOne™ Real-Time PCR System (Applied Biosystems, Monza, Italy) and StepOne software v 2.0 according to the manufacturer instructions. Designed primer pairs used to target selected *Chloroflexi* were: (i) 682f (5′-AGGCGAAAGCGGTTTCCAA-3′) and 814r (5′-ACTTAAAGCGTTAGCTTCGGCA-3′) for VLD-1; (ii) 585f (5′- TCAACTGGGAGGAGTCATTCG-3′) and 697r (5′- GAAACAGCCTAGAAAACCGCC-3′) for VLD-2 (Additional file 4: Table S2). Finally, known primers 920f [54] and 1044r [55] were used to target bacterial 16S rRNA genes. PCR cycles were as follows: (i) 95 °C for 10 min, (ii) 40 cycles of denaturing at 95 °C for 30 s, annealing at 56, 59 or 54 °C for 30 s (with uncultured *Chloroflexi* VLD-1, uncultured *Chloroflexi* VLD-2 and total bacterial 16S rRNA gene primer pairs, respectively), and elongation at 72 °C for 30 secs, (iii) denaturation at 95 °C for 15 s followed by a melting curve from 60 to 95 °C and fluorescence measure every 0.3 °C. The qPCR reactions (25 µL) were set-up as follows: 1× Power SYBR^®^ Green PCR Master Mix (Applied Biosystems, Monza, Italy), forward and reverse primers at 350 nM each and 2.5 µL of DNA template. 5-point standard curves were included in each plate from 6.8 × 10^3^ to 6.8 × 10^8^ copy numbers using PCR products of DGGE gel-purified bands as standard template for *Chloroflexi*-specific targets or of *E. coli* 16S rRNA gene for total bacteria. DNA was purified with Wizard^®^ SV Gel and PCR Clean-Up System (Promega Italia S.r.l, Milano, Italy) according to the manufacturer protocol and quantified with a P330 Nanophotometer (Implen GmbH, Munich, Germany). Amplification efficiencies ranged from 85 to 106%, with R^2^ = 96–100%. Samples and standards were set up in triplicate reactions. Genes copy numbers per gram of sediment were calculated and relative abundance was determined as percentage ratio between the copy number of target *Chloroflexi* 16S rRNA genes and the copy number of total bacterial 16S rRNA genes obtained from the same template DNA.

## Additional files

**Additional file 1: Table S1.** PCB total concentration and congeners distribution of the samples used in this study.

**Additional file 2: Figure S1.** Sulfate-reduction activities in dechlorinating and non-dechlorinating cultures throughout incubation.

**Additional file 3: Figure S2.** Methanogenic activities in dechlorinating and non-dechlorinating cultures throughout incubation.

**Additional file 4: Table S2.** Features of primer pairs designed in this study. The primers target specifically VLD-1 and VLD-2 16S rRNA genes. The table report sequence, melting temperatures and *in silico* specificity analyses.
