# Supplementary material for: Identification of two organohalide-respiring Dehalococcoidia associated to different dechlorination activities in PCB-impacted marine sediments
Source: Microb Cell Fact. 2017 Jul 24;16:127. doi: 10.1186/s12934-017-0743-4 (PMC5525228; doi:10.1186/s12934-017-0743-4)
Supplement: Supplementary file 1 — Additional file 1: Table S1. PCB total concentration and congeners distribution of the samples used in this study. [file 12934_2017_743_MOESM1_ESM.pdf]

Table S1: PCB total concentration and congeners distribution of the sediment samples used in this study. Values are the mean (± SD) of replicate analysis on three sediment subsamples.

**Sediment**	**Total PCB concentration** *(mg PCBs (kg dry sediment)$^{-1}$)*	**Di- and tri-chlorinated congeners** (wt %)	**Tetra- and penta-chlorinated congeners** (wt %)	**Hexa- and hepta-chlorinated congeners** (wt %)	**Octa- and nona-chlorinated congeners** (wt %)
**A**	0.4 ± 0.1	0.26%	28.08%	5.02%	66.64%
**B**	1.5 ± 0.8	0.00%	0.75%	5.96%	93.29%
**C**	1.3 ± 0.2	0.02%	1.52%	3.13%	95.33%
**D**	3.3 ± 2.8	0.00%	0.91%	27.27%	71.81%
**E**	0.6 ± 0.1	0.00%	3.00%	6.66%	90.34%
**F**	0.2 ± 0.0	0.00%	5.11%	10.36%	84.54%
