# Supplementary material for: Identification of two organohalide-respiring Dehalococcoidia associated to different dechlorination activities in PCB-impacted marine sediments
Source: Microb Cell Fact. 2017 Jul 24;16:127. doi: 10.1186/s12934-017-0743-4 (PMC5525228; doi:10.1186/s12934-017-0743-4)
Supplement: Supplementary file 4 — Additional file 4: Table S2. Features of primer pairs designed in this study. The primers target specifically VLD-1 and VLD-2 16S rRNA genes. The table report sequence, melting temperatures and in silico specificity analyses. [file 12934_2017_743_MOESM4_ESM.pdf]

Table S2: Features of primer pairs designed to target the 16S rRNA genes of VLD-1 and VLD-2 phylotypes.

**Target microorganism**	**Primer**			**Product length**	**Non-target products** *(per mismatch)*		
	Name	*Sequence (5'→3')*	*Tm (°C)*	*(bp)*	*0*	*1*	*2*
Uncultured *Chloroflexi* VLD-1	682f	AGGCGAAAGCGGTTTCCAA	60.5	153	0	4	26
	814r	ACTTAAAGCGTTAGCTTCGGCA	60.3				
Uncultured *Chloroflexi* VLD-2	585f	TCAACTGGGAGGAGTCATTCG	59.5	133	0	6	11
	697r	GAAACAGCCTAGAAAACCGCC	59.8				
